# Annotation-based genome-wide SNP discovery in the large and complex *Aegilops tauschii *genome using next-generation sequencing without a reference genome sequence

**DOI:** 10.1186/1471-2164-12-59

**Published:** 2011-01-25

**Authors:** Frank M You, Naxin Huo, Karin R Deal, Yong Q Gu, Ming-Cheng Luo, Patrick E McGuire, Jan Dvorak, Olin D Anderson

**Affiliations:** 1 Department of Plant Sciences, University of California, Davis, CA, 95616, USA; 2 Genomics and Gene Discovery Research Unit, USDA-ARS, Western Regional Research Center, Albany, CA, 94710, USA

## Abstract

**Background:**

Many plants have large and complex genomes with an abundance of repeated sequences. Many plants are also polyploid. Both of these attributes typify the genome architecture in the tribe Triticeae, whose members include economically important wheat, rye and barley. Large genome sizes, an abundance of repeated sequences, and polyploidy present challenges to genome-wide SNP discovery using next-generation sequencing (NGS) of total genomic DNA by making alignment and clustering of short reads generated by the NGS platforms difficult, particularly in the absence of a reference genome sequence.

**Results:**

An annotation-based, genome-wide SNP discovery pipeline is reported using NGS data for large and complex genomes without a reference genome sequence. Roche 454 shotgun reads with low genome coverage of one genotype are annotated in order to distinguish single-copy sequences and repeat junctions from repetitive sequences and sequences shared by paralogous genes. Multiple genome equivalents of shotgun reads of another genotype generated with SOLiD or Solexa are then mapped to the annotated Roche 454 reads to identify putative SNPs. A pipeline program package, AGSNP, was developed and used for genome-wide SNP discovery in *Aegilops tauschii-*the diploid source of the wheat D genome, and with a genome size of 4.02 Gb, of which 90% is repetitive sequences. Genomic DNA of *Ae. tauschii *accession AL8/78 was sequenced with the Roche 454 NGS platform. Genomic DNA and cDNA of *Ae. tauschii *accession AS75 was sequenced primarily with SOLiD, although some Solexa and Roche 454 genomic sequences were also generated. A total of 195,631 putative SNPs were discovered in gene sequences, 155,580 putative SNPs were discovered in uncharacterized single-copy regions, and another 145,907 putative SNPs were discovered in repeat junctions. These SNPs were dispersed across the entire *Ae. tauschii *genome. To assess the false positive SNP discovery rate, DNA containing putative SNPs was amplified by PCR from AL8/78 and AS75 and resequenced with the ABI 3730 xl. In a sample of 302 randomly selected putative SNPs, 84.0% in gene regions, 88.0% in repeat junctions, and 81.3% in uncharacterized regions were validated.

**Conclusion:**

An annotation-based genome-wide SNP discovery pipeline for NGS platforms was developed. The pipeline is suitable for SNP discovery in genomic libraries of complex genomes and does not require a reference genome sequence. The pipeline is applicable to all current NGS platforms, provided that at least one such platform generates relatively long reads. The pipeline package, AGSNP, and the discovered 497,118 *Ae. tauschii *SNPs can be accessed at (http://avena.pw.usda.gov/wheatD/agsnp.shtml).

## Background

Single nucleotide polymorphisms (SNPs) are valuable markers for the construction of genetic and physical maps, genome sequencing, marker-assisted selection, and for other genetic and genomic applications. Resequencing is the most efficient approach for a large scale, genome-wide SNP discovery. For example, resequencing with the Sanger sequencing technology using an annotated genome sequence as a reference has been an effective strategy for genome-wide SNP discovery in low or moderately complex genomes [1]. Compared to Sanger sequencing, massively parallel sequencing technologies, such as the 454 GS FLX Instrument (Roche Applied Science), Solexa (Illumina Inc), and SOLiD (Life Technologies Inc), offer high sequencing throughputs at greatly reduced costs. Each of these sequencing platforms has its own set of advantages and disadvantages. Roche 454 generates longer sequences (200-500 bp or more, depending on the version of the platform) than Solexa (35-150 bp) or SOLiD (25-75 bp), but SOLiD and Solexa have higher throughputs than Roche 454 with the same cost and time investment. Greatly enhanced throughput at reduced cost and time investment is common to all next-generation sequencing (NGS) platforms and allows for deep genome coverage sequencing, a prerequisite for genome-wide SNP discovery in the complex genomes of plants and animals.

Many plants have large and complex genomes with a great abundance of repeated sequences. Polyploidy, a frequent evolutionary strategy in the plant kingdom, further increases genome size and complexity. These attributes are common in the tribe Triticeae, which includes such economically important plants as wheat, barley and rye. These features of the Triticeae genomes present a formidable challenge to genome-wide SNP discovery with NGS platforms, primarily because the abundance of highly repetitive sequences makes alignment and clustering of the short reads generated by some of the NGS platforms difficult.

Strategies are available to ameliorate these difficulties. Reduced representation libraries (RRLs) include only a subset of sequences present in a complex genome. The RRL subset is then used for resequencing, sequence alignment, assembly, and SNP discovery [1,2]. In plants, RRLs have been used for SNP discovery in maize [3], rice [4], soybean [4,5], and common bean [6]. The use of cDNA libraries for NGS is another and frequently used approach to reduce complexity, avoid repetitive sequences and target coding sequences for SNP discovery. Deep transcriptome resequencing with NGS platforms has been used for SNP discovery in maize [7] and the polyploid *Brassica napus *[8]. While both strategies can dramatically reduce sequence complexity, each has limitations. For example, transcriptome sequencing ignores polymorphism in introns and other genic regions absent from mRNA.

An undesirable feature of transcriptome resequencing for SNP discovery is unavailability of potentially useful transposable element (TE)-derived polymorphisms. In contrast to SNPs embedded within repeated sequences (which are notoriously difficult as markers), the junctions of neighbouring repeated sequences are potentially unique [9-11], can be assayed [10-12] and SNPs in them can be treated as single-copy markers. Repeat junctions (RJs) are created by insertions of TEs into each other, into genes, or into other DNA sequences [9,10]. A high-throughput assay for RJ markers has been reported [9,11]. Because SNPs at RJs are dispersed over a whole genome, they are well suited for the construction of dense, genome-wide SNP genetic maps. An important application of such maps is in the anchoring and the ordering of contigs of bacterial artificial chromosome (BAC) clones during the construction of physical maps or ordering of scaffolds during genome sequencing. To use RJs in SNP discovery, dedicated annotations of NGS reads are required and relevant computational tools for RJ identification have been developed [10,11].

A genomic sequence serving as a reference has been the basis of genome-wide SNP discovery utilizing NGS technologies. Tremendous progress has been achieved in the development of algorithms and software tools for mapping short reads from different NGS platforms to a reference genome and then identifying variants between individual sequences and the reference genome sequences [13-16]. However only a few attempts have been reported utilizing NGS to discover SNPs when such a reference is unavailable [5]. A computational pipeline called DIAL (*de novo *identification of alleles) was recently released for the identification of SNPs between two closely related genotypes without the help of a reference genome sequence [17]. This tool first masks repetitive sequences and then clusters short reads from the genotypes. The clustered reads are assembled with a *de novo *assembler to identify variants. This tool can be used for the clustering of reads of genome and transcriptome sequences from Roche 454 and Solexa even with a shallow depth of genome coverage. However, the tool has been tested only on relatively low- or moderately-complex genomes. It does not allow SNP discovery in repeat junctions and is applicable only to base-space reads, such as with Roche 454 and Illumina Solexa, but is not applicable to the two-base encoded, color space SOLiD reads.

We report here an annotation-based genome-wide SNP discovery pipeline using NGS data for complex genomes without a reference genome sequence. In this pipeline, Roche 454 shotgun reads with low genome coverage of one genotype are annotated to distinguish single-copy reads covering genes, repeat junctions and other sequences from repetitive sequences and paralogous gene sequences. The annotation dramatically reduces the complexity of the genomic sequences by removing undesirable sequences. Resulting reads are assembled into sequence contigs if possible. The assembled Roche 454 contig and singleton sequences mimic a reference sequence. Shotgun short reads of another genotype with high genome coverage generated with the SOLiD or Solexa NGS platforms are then mapped to the annotated single-copy Roche 454 reads/contigs to identify SNPs in single copy DNA across the entire genome including repeat junctions. Based on this strategy, the pipeline program package, AGSNP, was developed and used for SNP discovery between two accessions of *Ae. tauschii *(AL8/78 and AS75), the parents of the F_2 _mapping population used for the construction of an *Ae. tauschii *genetic map [18]. *Aegilops tauschii *contains the core genome of the Triticum-Aegilops alliance [19] and is the diploid source of the wheat D genome [20,21]. Its genome size is 4.02 Gb [22] and 90% of its genome is composed of repetitive sequences [23]. It is also an important source of germplasm in wheat breeding and a diploid model for the wheat D-genome.

## Methods

### Next-generation sequencing

In order to test SNP discovery efficiency in different sequencing platforms, three next-generation sequencing platforms (Roche 454, Illumina Solexa and ABI SOLiD) were used to sequence two *Ae. tauschii *genotypes (AL8/78 and AS75) (Table 1). Genomic DNA of AL8/78 was sequenced using only the Roche 454 whereas genomic DNA of AS75 was sequenced using all three NGS platforms. In addition, cDNA of AS75 was also sequenced using the ABI SOLiD platform to verify SNPs identified in genic regions.

**Table 1:** Next generation sequences used for SNP discovery and for estimation of sequencing error rates

Accession	Sequencing platform	Sequence type	Total reads	Total size (Mb)	Average Read length (bp)	Genome coverage
AL8/78	Roche 454 GS-FLX Titanium	Shotgun genomic	14,087,315	5,445	380.5	~1.35X^(a)^

AS75	Roche 454 GS-FLX Titanium	Shotgun genomic	1,394,433	433	310.8	~0.11X

AS75	Illumina Solexa	Shotgun genomic	74,814,052	6,284	84	~1.56X

AS75	AB SOLiD v3.0	Shotgun genomic	2,136,678,966	106,834	50	~26.57X

AS75	AB SOLiD v3.0	Shotgun cDNA	442,086,124	22,104	50	~22X^(d)^

AL8/78	Roche 454 GS-FLX Titanium	13 BACs^(b)^, genomic	58,971	24.7	418.9	~12.93X

AL8/78	Illumina Solexa	13 BACs, genomic	5,586,903	223	40	~116.75X

AL8/78	AB SOLiD v2.0	13 BACs, genomic	26,013,814	624	25^(c)^	~326.87X

(a) Genome sizes were estimated based on the 4.02 Gb genome size of *Aegilops tauchii *[22]. (b) The total size of the 13 BACs is 1.91 Mb. (c) Only 24 bases were used for mapping. (d) Genome coverage was estimated on the basis of average coverage depth of SOLiD cDNA reads mapping to AL8/78 shotgun reads.

To estimate sequencing error rates intrinsic to each platform and variant calling errors in different sequencing platforms, DNAs of 13 *Ae. tauschii *(AL8/78) BAC clones were separately fragmented and shotgun sequenced with an ABI 3730 xl (henceforth Sanger sequence). These Sanger sequences were used as a reference in the estimation of sequencing error rates. Pooled DNAs of the 13 *Ae. tauschii *BAC clones were sequenced on three platforms to depths ranging from 12.9X for Roche 454 to 326.9X for SOLiD (Table 1).

#### Roche 454 sequencing

Preparation and sequencing of the 454 sequencing library was performed according to the manufacturer's instructions (GS FLX Titanium General library preparation kit/emPCR kit/sequencing kit, Roche Diagnostics, http://www.roche.com). In brief, ten micrograms of *Ae. tauschii *genomic DNA or pooled DNA of 13 *Ae. tauschii *BACs were sheared by nebulization and fractionated on agarose gel to isolate 400-750 base fragments. These were used to construct a single-stranded shotgun library that was used as a template for single-molecule PCR. The amplified template beads were recovered after emulsion breaking and selective enrichment. The Genome Sequencer FLX Titanium flows 200 cycles of four solutions containing either dTTP, αSdATP, dCTP and dGTP reagents, in that order, over the cell.

#### Illumina Solexa sequencing

The AS75 library of genomic DNA or the AL8/78 library of pooled DNA of 13 BACs was quantified by analysis on an Agilent Bioanalyzer (Agilent Technologies, Inc.), using the instrument software to select a region comprising the main library peak. Based on the calculated value, the library was applied to an Illumina single read flow cell at 5 pM concentration and clusters were generated according to manufacturer's instructions. Sequencing was carried out on an Illumina Genome Analyzer GAIIX for 85 cycles. Two, version 3, 36 cycle, kits were used. Data was generated following completion of the run using the Illumina Pipeline 1.4 from the sequencing images. A phix control lane was used to generate phasing and matrix values that were then applied to the experimental samples for basecalling analyses.

#### ABI SOLiD sequencing

DNA was isolated from nuclei of *Ae. tauschii *accession AS75 as described in Dvorak et al. 1988 [24]. A fragment library was constructed according to manufacturers' instructions using the Applied Biosystems Fragment Library Construction Kit (Life Technologies, Inc.). In brief, 5 ug of DNA was sheared using the CovarisTM S2 system (Covaris, Inc.), the sheared DNA was end-repaired, adaptors P1 and P2 were ligated to the end-repaired DNA, and the DNA was size-selected on a gel. The size-selected DNA was nick translated and then amplified for 3 cycles to generate the fragment library. The fragment library was quantified using the Agilent DNA high-sensitivity kit on an Agilent 2100 Bioanalyzer (Agilent Technologies, Inc.).

To construct a cDNA library, *Ae. tauschii *line AS75 was grown in a solution culture containing 0.5X Hoagland solution and total RNA was isolated from both roots and shoots according to manufacturer's instructions using the Ambion RNAqueous kit and the Ambion Plant RNA Isolation Aid (Life Technologies, Inc.). mRNA was isolated from total RNA according to manufacturer's instructions using the Applied Biosystems Poly(A) Purist Kit (Life Technologies, Inc.). The transcriptome library was constructed according to manufacturers' instructions using the Whole Trascriptome Anaysis Kit from Applied Biosystems (Life Technologies, Inc.). In brief, mRNA was fragmented using RNase III and size-selected. The size-selected RNA was reverse transcribed, and the cDNA size selected. The size-selected cDNA was amplified using 15 cycles to create the transcriptome library. This library was quantified using the Agilent DNA high-sensitivity kit on an Agilent 2100 Bioanalyzer (Agilent Technologies, Inc.). The root and shoot cDNAs were combined.

Templated beads were prepared from both the fragment library and the transcriptome library according to manufacturer's instructions using the ePCR kit v.2 and the Bead Enrichment Kit from Applied Biosystems (Life Technologies, Inc.) for SOLiD3. Workflow Analysis was done after the first round of templated bead preparation for each library according to manufacturer's instructions using the Workflow Analysis kit from Applied Biosystems (Life Technologies, Inc.) to check library quality and the amount of templated beads generated per ePCR. An additional Workflow Analysis was done for both libraries after it was estimated that a sufficient number of templated beads were produced. Templated beads were deposited on slides according to manufacturers' instructions using the Bead Deposition kit from Applied Biosystems (Life Technologies, Inc.). One full slide was run for the transcriptome library, while 5 full slides (2.5 full runs) were run for the fragment genomic library.

### Sequencing errors of NGS platforms and variant calling error

#### Single read based sequencing errors

The single read sequencing error rate of the Roche 454 GS-FLX Titanium platform was estimated by comparing the single read alignment of AL8/78 reads in a pool of 13 BAC clones previously sequenced with the Sanger method. Because Sanger BAC sequences were based on the shotgun sequencing method they had a negligible error rate. Alignments were obtained using BLASTN of Roche 454 reads against 13 AL8/78 BAC sequences. Insertion and deletion (INDEL), and single base substitutions were counted. The sequencing error rate was calculated as total erroneous bases divided by the total length in bp of Roche 454 reads.

#### Consensus-based sequencing errors

AL8/78 reads from Roche 454, SOLiD or Solexa were mapped to the 13 BAC sequences generated by the Sanger method using the bwa package [15,16] at default parameters and consensus sequence of mapped reads were generated using SAMTools [25]. INDEL and single substitutions were counted by comparing Sanger sequences and mapped read consensus sequences. A consensus sequencing error rate of a sequencing platform was calculated as the total erroneous bases divided by the total mapped bases.

#### Variant calling errors based on Roche 454 single reads as a reference sequence

AL8/78 reads from SOLiD, Solexa or Roche 454 were mapped to Roche 454 genomic contigs or singletons using the bwa package [15,16] at default parameters. Consensus sequences of mapped reads were generated using SAMtools [25]. The same method was used to count INDEL errors and single-base substitutions and to calculate variant calling error rates.

### SNP discovery pipeline

#### Rationale and strategy

Genome-wide SNP discovery involves two basic steps: (1) the alignment of sequences of two or more genotypes and (2) variant calling in the aligned sequences. Alignment of NGS on a reference genome sequence is called read mapping to the reference sequence. When a reference genome sequence is available, even short reads can be relatively easily mapped and aligned for the purpose of variant calling. In the absence of a genome sequence, long reads (such as those produced by Sanger or Roche 454 sequencing) from different genotypes can be clustered and aligned via multiple alignment algorithms [26]. Difficulties emerge if no reference sequence is available, especially if only short reads generated by the SOLiD or Solexa sequencing platforms are available and genome is highly repetitive. In the strategy used here and similarly reported by Hyten et al. (2010) [6], the relatively long Roche 454 reads are substituted for the reference genome sequence. Roche 454 reads are annotated, i.e., they are classified on the basis of their sequence homology and copy number in the genome. Single-copy sequences and unique repeat junction sequences are subsequently used as a reference sequence for the alignment of the SOLiD or Solexa reads and for SNP discovery.

The following rationale is used to identify (annotate) Roche 454 single-copy sequences. It is assumed that most genes are in a single-copy dose in a genome and sequences of duplicated genes are usually diverged to such an extent that most of their reads do not cluster together. Therefore, the read depth (number of reads of the same nucleotide position) mapped to coding sequences of known genes estimates the expected read depth of all single-copy sequences in a genome. Sequences showing greater read depth are assumed to be from duplicated or repeated sequences. To implement this rationale, shallow genome coverage by long Roche 454 sequences is used to identify genic sequences by homology search against gene databases. Multiple genome coverages of short SOLiD or Solexa sequences are then used to estimate the read depth of genic sequences in a population of SOLiD or Solexa reads. The estimate is in turn used to identify (annotate) the remaining single-copy Roche 454 reads. This combination of Roche 454 and SOLiD or Solexa platforms combines the long length of Roche 454 reads with the high coverage of the SOLiD/Solexa sequencing platforms, thus reducing costs associated with the development of reference sequence, as already pointed out by Hyten et al. (2010) [6]. Short SOLiD or Solexa reads are mapped and aligned to the Roche 454 reads and contigs with short-read mapping tools [13-16,25]. After the annotation of all sequences, SNPs are called and filtered.

#### Annotation of Roche 454 reads

**Figure 1:**
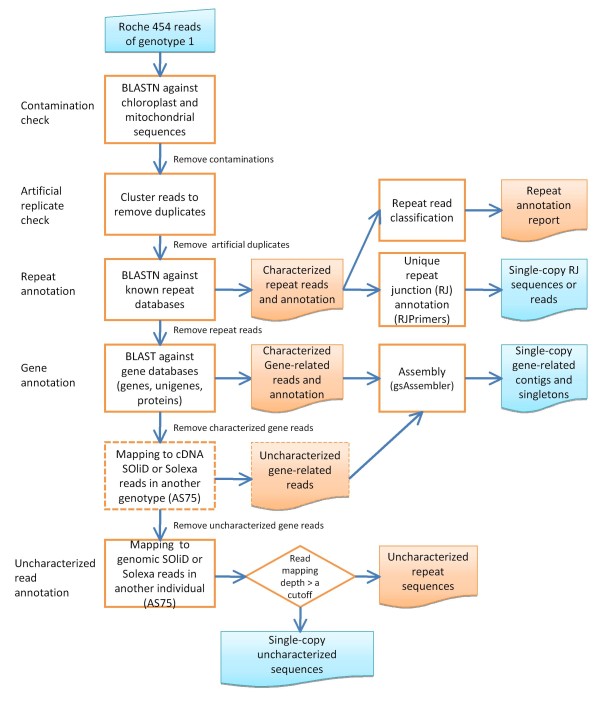
**Annotation pipeline of Roche 454 reads from the *Ae. tauschii *accession AL8/78 (genotype 1)**. Predicted single-copy gene-related sequences, uncharacterized sequences and repeat junction sequences will be used for SNP discovery. The processes with dashed boxes are optional depending on whether or not cDNA short reads are available.

The aim of read annotation is to classify the Roche 454 reads on the basis of their homology and copy number. For annotation of a large volume of Roche 454 shotgun reads from multiple runs, a substantial amount of time and computer resources is required. Annotation was therefore divided into several steps, and a corresponding program was developed for each step. Figure 1 and Table S1 in Additional file 1 show the annotation pipeline and the corresponding pipeline script programs, respectively.

#### Pre-processing of Roche 454 reads of Ae. tauschii accession AL8/78

The purpose of pre-processing of reads is to remove the chloroplast and mitochondrial sequences. BLASTN against complete wheat chloroplast and mitochondrial genome sequences (AB042240 and AP008982) was performed at an E value of 1E-10. A total of 14,087,315 Roche 454 reads of the AL8/78 genomic library were processed. After the removal of chloroplast and mitochondrial reads, artificial replicates of reads were filtered out using the cd-hit-454 program [27] at 98% alignment identity and 90% sequence coverage. Artificial replicates are intrinsic artifacts of 454-based pyrosequencing occurring in all currently available 454 technologies, leading to overpresentation of >10% of the original DNA sequencing templates. Those sequences start at the same position and are identical (duplicates) or vary in length, or contain a sequence discrepancy [28,29].

#### Characterized-repeat annotation

The first annotation step is to identify reads of known (characterized) repeats. To extract all characterized repetitive reads, all possible plant repeat databases used in RJPrimers [10], including RepBase14.07, MIPS REdat v4.3 [30], the complete TREP (release 10), the maize transposable element database (maize TEDB) (July 2009), and 12 TIGR repeat databases [31], were adopted to perform a BLAST search at an E-value of 1E-10. The extracted characterized repeat reads were further annotated using the repeat junction annotation pipeline (Table S1 in Additional file 1) to identify unique RJs, which are used for SNP identification.

#### Gene annotation

Homology search against known genes is a fundamental approach to identify genes among the sequences generated. The reads remaining after removing repetitive reads were used to search for homology against databases of genes, proteins and unigenes in all species evolutionally related to the targeted genome, *Ae. tauschii*, including the following: complete genome gene databases of *Brachypodium *(v1.0), rice (RAP-DB) (build 5) [31-33], sorghum (bicolor-79), and maize; the unigene database of wheat (build #57), rice (build #82), sorghum (build #29), sugarcane (build #14), barley (build #56), maize (build #71) and *Arabidopsis *(build #79); the UniProt protein database (plant only, release 2010-07), and the *Brachypodium *protein database (v1.0). An E value of 1E-10 was used for both BLASTN and BLASTX searches. Reads related to transposable elements existing in protein or unigene databases were also removed. Some unknown gene reads were further identified using SOLiD cDNA reads of AS75 (~22X gene coverage) mapping to the Roche 454 reads of AL8/78 with the pipeline program (bwa_mapping_pipeline.pl, Table S1 in Additional file 1). All known and uncharacterized gene reads of Roche 454 were assembled at a higher stringency (95% of alignment identity) using gsAssembler (Roche Applied Science) (batch_gsassembly.pl, Table S1 in Additional file 1).

#### Single-copy read annotation

**Figure 2:**
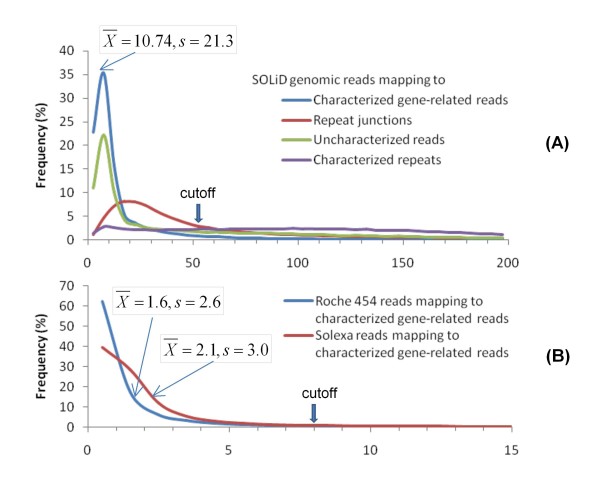
**Frequency distributions of the depths of NGS reads of AS75 mapped to annotated Roche 454 reads**. Except that for characterized repeat reads, the remaining distributions can be approximated to an extreme value distribution. (A) Frequency distributions of the depths of AS75 SOLiD genomic reads (total ~ 26X genome equivalents) mapped to Roche 454 characterized gene reads, repeat junction reads, characterized repeat reads, and uncharacterized reads. Because most gene-related reads are single copy, the frequency distribution of reads mapped to gene-related reads is used as single-copy read distribution. The estimated population mean ($\bar{X}$) plus two standard deviations (*s*) (depth of 53X) of this distribution was used as the cut-off depth for considering AS75 SOLiD genomic reads mapped on Roche 454 AL8/78 gene reads, repeat junctions, and uncharacterized reads as single-copy. (B) Frequency distributions of read depths and $\bar{X}$ + 2*s *cut-off values for Solexa AS75 genomic reads (~1.56X genome equivalent) and Roche 454 AS75 genomic reads (~0.11X genome equivalent) mapped to characterized gene reads of Roche 454. The distributions were skewed to the left because of low coverage but still could be fitted to an extreme value distribution (a Weibull distribution) [34].

After the removal of characterized and uncharacterized gene reads, the remaining Roche 454 reads consist of uncharacterized repeats and other uncharacterized sequences, including introns, promoters, pseudogenes, unknown genes, and single-copy sequences in intergenic spaces. Since many of these sequences are single-copy, they are desirable targets for SNP discovery. They were identified on the basis of the rationale described above. SOLiD or Solexa reads of *Ae. tauschii *accession AS75 were mapped to known gene reads obtained in the gene annotation routine using the mapping pipeline (bwa_mapping_pipeline.pl, Table S1 in Additional file 1). A depth frequency distribution of SOLiD or Solexa reads mapped to characterized gene reads approximates an extreme value distribution [34] (Figure 2A). This distribution is used to estimate the single-copy read depth of SOLiD or Solexa sequences. Mean ($\bar{X}$) and standard deviation (*s*) of mapped read depth are calculated from the fitted extreme value distribution. The $\bar{X}$ + 2*s *of read depths is used as a cut-off value for single-copy sequences. If a read depth is < the cut-off value, the reads are considered to be single-copy sequences; if it is > the cut-off value, they are assumed to be repeated. For instance, the mapping of AS75 SOLiD genomic reads (~26X genome equivalent) to Roche 454 characterized gene reads generated a depth distribution with an $\bar{X}$ of 10.7 and an *s *of 21.3 (Figure 2A). Thus, the read depth cut-off value was 53 reads. Roche 454 sequences with depth < 53 reads were considered single-copy. Approximately 77% of characterized gene reads or 92% of assembled gene contigs/singletons were included in this single-copy class. Remaining gene reads may be duplicated genes. Reads from other sequencing platforms were separately mapped to known Roche 454 gene reads and empirical distributions of mapped read depths were generated for each sequencing platform (Figure 2B). A read depth cut-off value of 8 reads was used for Solexa and Roche 454 genomic reads (Figure 2B). These cut-off values were used as a criterion for annotation of single-copy gene sequences and repeat junctions among the total reads produced by the various platforms for subsequent SNP discovery.

#### SNP discovery

**Figure 3:**
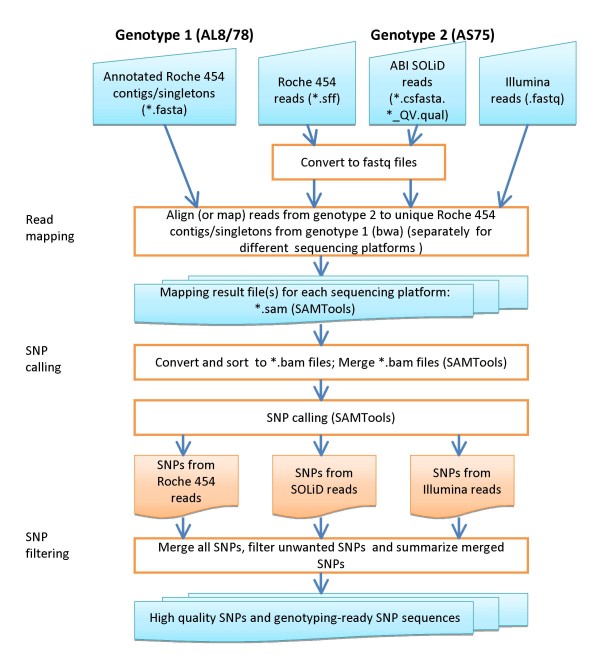
**SNP discovery pipeline using Roche 454 reads of the *Ae. tauschii *accession AL8/78 as a reference**.

The SNP discovery procedure is diagrammed in Figure 3. Reads mapped to Roche 454 annotated contigs or singletons (single reads) were analyzed separately for the different sequencing platforms. The mapping tool bwa [15,16] is employed because of its suitability for mapping either long reads such as Roche 454 and Sanger or short reads (SOLiD and Solexa). All possible variants including insertions/deletions (INDEL) and SNPs, are then called from aligned sequences using the SAMTools program [25]. Because most sequencing errors in Roche 454 arise from homopolymers that result in INDEL errors (see Results), short INDEL variants are considered erroneous if Roche 454 sequences are used as a reference. Therefore, only single nucleotide substitutions are considered SNPs in the pipeline.

#### SNP filtering criteria

To filter out potentially false positive SNPs, additional filtering criteria are imposed on SNPs called by SAMTools in the pipeline program "snp_filter_pipeline.pl" (Table 2 and Table S1 in Additional file 1). Long Roche 454 reads or contigs (≥ 200 bp) are beneficial to primer design for SNP genotyping. A minimum read depth of 3 reads is used to minimize false alignments of reads on the reference sequence. The mapped read depth cut-off value is a critical criterion to filter out SNPs located in repetitive sequence and paralogous genes. The consensus base ratio is the number of reads to the total number reads derived from a single DNA source mapped to a reference sequence having a nucleotide that differs from the corresponding nucleotide in the reference sequence. If the consensus base ratio is 1.0, there is a high confidence in the SNP. If the ratio is < 1.0, some reads have the same nucleotide as the reference sequence, and there is less confidence in the SNP. Such SNPs are either caused by heterozygosity of the DNA source or by a sequencing error. Thus, a highly stringent criterion (≥0.9) is imposed to exclude heterozygous loci or false positive SNPs due to alignment errors.

**Table 2:** SNP filtering criteria used in this study

	Item	Criteria of putative SNPs
1	Reference sequence length	≥ 200 bp

2	Minimum mapped read depth to the reference	≥ 3
	
	Maximum mapped read depth to the reference	Roche 454: ≤ 5 Solexa:≤ 10 SOLiD genomic reads: ≤ 50 SOLiD cDNA: ≤ 100

3	Consensus base ratio	≥ 0.9

4	Mapping quality score in SAMTools	≥ 20
	
	Reference SNP base quality score and neighborhood quality standard (NQS) score	SNP base ≥30 NQS 11 bases: ≥ 20

5	Removing homopolymer SNPs	SNP base string length ≥ 3 bp

6	Removing very close SNPs	> 3 bp between two contiguous SNPs

7	Removing SNPs at the right side of 454 reads	> 30 bp away from the right side

8	Illumina genotyping quality (optional for SNP discovery but recommended for SNPs intended for Illumina GoldenGate assays )	≥ 60 bp between two contiguous SNPs

**Figure 4:**
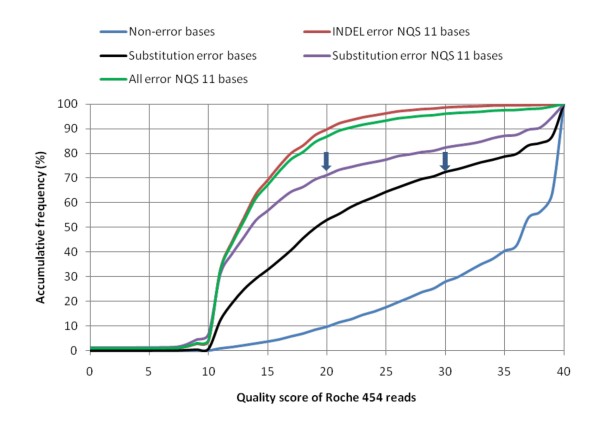
**Roche 454 sequencing errors in relation to base quality score of reads**. Over 70% of base substitution errors (arrows) can be filtered out if the SNP base quality score is ≥ 30 and the neighbourhood quality standard (NQS) 11 base score is ≥ 20.

Two types of quality scores are used for reference sequences and mapped reads. A mapping quality score is provided by the bwa tool [15,16,25] to measure the mapping quality of short reads aligned to a reference sequence; 20 is suggested for high quality mapping [15,16,25]. For the reference sequences (Roche 454 reads here), a SNP base quality score (≥ 30) and a neighbourhood quality standard (NQS) 11 base score (≥ 20) are applied which together can filter out over 70% of substitution sequence errors (Figure 4). Homopolymer-induced sequence errors are the major error source in Roche 454 sequencing, accounting for 85% of all errors. Thus, all SNPs with the same base string longer than or equal to 3 bp are removed. In addition, if two SNPs are separated by 3 bp or less, then most of them are assumed to be caused by incorrect alignments or short reads mapped on a wrong reference sequence. Those SNPs are also filtered out.

**Figure 5:**
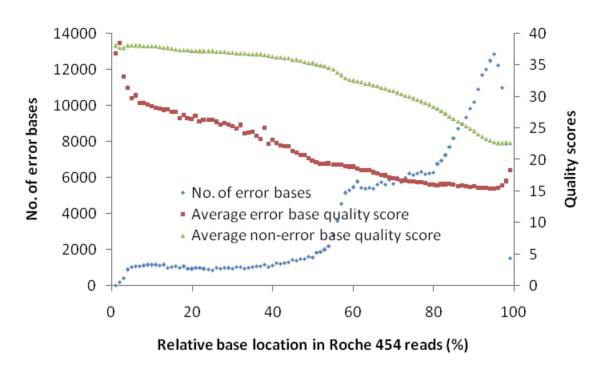
**The relationship of Roche 454 sequencing errors to relative error locations in reads**.

Sequence quality deteriorates with the length of a Roche 454 read. Hence, there is less confidence in SNPs on the 3' end of a reference sequence than on the 5' end (beginning). Therefore, the quality of bases is related to their relative location in a read (Figure 5). We found a significant correlation between base location in a read and number of error bases (r^2 ^= 0.87, p < 0.0001) in Roche 454 sequences. The 3' end bases of a read have an increasingly higher error rate. Thus, SNPs with ≤30 bp on the 3' end of single reads or contigs are ignored.

In addition, Illumina's GoldenGate or Infinium assays require a minimum of 50 bp (60 bp preferred) of sequence on either side of each SNP and a minimum of 60 bp between two contiguous SNPs. These requirements for Illumina genotyping are optionally applied in the pipeline program.

More stringent SNP filtering criteria are imposed for uncharacterized reads because most of them should be unknown low-copy repetitive sequences. We set the maximum mapped read depth cut-off to 5 reads instead of 8 for both Roche 454 and Solexa reads, and 25 instead of 53 for SOLiD with the aim to eliminate SNPs in potentially low-copy repetitive sequences. In addition, reads with only one SNP are retained.

All annotated AL8/78 single-copy gene-related sequences, RJs, and uncharacterized single-copy sequences were used as a reference sequence in mapping reads from Roche 454, Illumina Solexa and ABI SOLiD of *Ae. tauschii *accession AS75 (Table 1). Variants (short INDEL and SNP) were called using the read mapping and SNP calling pipeline (bwa_snp_pipeline) with the bwa [15,16] and SAMTools package [25]. All called variants from different sequencing platforms and DNA sources (genomic or transcriptome) were merged and filtered using the SNP filter pipeline program (summarize_bwa_snp_calls.pl and snp_filter_pipeline.pl, Table S1 in Additional file 1). All short INDELs were excluded and only high-quality SNPs were retained.

#### PCR validation

A total of 192 gene-related sequences, 96 repeat junction sequences and 95 uncharacterized sequences with at least one SNP were randomly selected among the identified SNPs for PCR validation. Primers flanking SNPs were designed with BatchPrimer3 [35]. DNA targets in both *Ae. tauschii *AL8/78 and AS75 were PCR amplified. Amplicons were sequenced using the PCR primers as sequencing primers with an ABI 3730 xl DNA Analyzer as described by Choi et al. (2007) [36].

## Results

### Estimation of NGS error rates

Sequencing errors may potentially be an important source of SNP errors, particularly since only 1.35X genome equivalents of Roche 454 sequences were used to construct the reference sequence. The single read error rate of Roche 454 was estimated to be 0.74%. Single read error rates could not be estimated for the other two platforms because of short reads. Consensus sequences generated from multiple read alignments can efficiently correct single read errors. Consensus error rates of Solexa and SOLiD appeared to be similar (0.043%-0.044%) and lower than the Roche 454 consensus error rate, which was 0.13% (Table 3). Errors of Roche 454 are primarily due to INDELs, which account for 75%-80% of all consensus sequence errors, while INDEL errors in Solexa and SOLiD reads accounted for 22% and 66% of all consensus sequence errors, respectively. After removing INDEL errors, the three NGS platforms appeared to have similar consensus base substitution error rates, 0.018%, 0.035%, and 0.030% for SOliD, Solexa, and Roche 454, respectively (Table 3). The low base substitution rate error of the SOLiD platform most likely reflected the di-base encoding and color space scheme in SOLiD sequencing technology.

**Table 3:** Sequencing and variant calling errors of next-generation sequencing based on the data set of Sanger sequences of 13 AL8/78 BAC clones

Error type	Platform	Overall error rate	INDEL error rate	Substitution error rate	Insertion (%)	Deletion (%)	Substitution (%)
Sequencing error compared with Sanger sequences							

Single read error	Roche 454 GS-FLX Titanium	7.4 × 10^-3^	6.2 × 10^-3^	1.2 × 10^-3^	42.41	41.93	15.66

Consensus error	Roche 454 GS-FLX Titanium	1.3 × 10^-3^	1.0 × 10^-3^	3.0 × 10^-4^	25.20	55.56	19.24
	
	Illumina Solexa	4.4 × 10^-4^	9.0 × 10^-5^	3.5 × 10^-4^	3.46	17.78	78.77
	
	AB SOLiD v2.0	4.3 × 10^-4^	2.5 × 10^-4^	1.8 × 10^-4^	10.69	47.58	41.73

Variant calling errors using Roche 454 sequence as reference							

Consensus error	Roche 454 GS-FLX Titanium	3.79 × 10^-3^	1.12 × 10^-3^	2.67 × 10^-3^	11.82	17.66	70.52
	
	Illumina Solexa	1.87 × 10^-3^	4.1 × 10^-4^	1.46 × 10^-3^	8.41	13.7	77.87
	
	AB SOLiD v2.0	5.9 × 10^-4^	2.7 × 10^-4^	3.2 × 10^-4^	21.71	24.67	53.62

To estimate SNP errors, a random half of Roche 454 reads constructed for the 13 *Ae. tauschii *BACs were used as references. Then SOLiD and Solexa BAC reads or another half of Roche 454 BAC reads were compared with Roche 454 reference sequences. In this case, errors on both sides contributed to error rates. The consensus base substitution error rates were 0.032%, 0.146% and 0.267% for SOLiD, Solexa and Roche 454, respectively (Table 3). Because SNPs with low quality scores (< 30 for SNP base and < 20 for NQS 11 bases) are filtered out in the SNP filtering pipeline (Table 2), ~70% of SNP errors can be eliminated (Figure 4). Therefore, much lower SNP error rates are expected (30% of the consensus base substitution error rate), about 0.01% for SOLiD, 0.04% for Solexa and 0.08% for Roche 454 (Table 3).

### Annotation of Roche 454 reads of *Ae. tauschii *accession AL8/78

A total of 14,087,315 Roche 454 reads was generated by shotgun sequencing of genomic DNA of *Ae. tauschii *accession AL8/78. The average read length was 380.5 bp and the genome coverage was 1.35X genome equivalents. After removing chloroplast and mitochondrial reads (1.64%), 13,856,244 reads were retained. A total of 1,570,944 reads (11.33%) was classified as artificial replicates detected with the cd-hit-454 software [27]. After their removal, 12,285,300 reads were retained (Table 4). BLAST searches against all available plant repeat databases [10] characterized a total of 59% of these reads as repeat reads. Among them, 4.9% of repeat reads were identified as containing repeat junctions using the repeat junction annotation pipeline with RJPrimers [10] (Table S1 in Additional file 1).

**Table 4:** Annotation and SNP discovery using Roche 454 reads of genomic DNA of *Ae. tauschii *AL8/78 as reference sequences

Category	**No. of reads **^**(a)**^	Length in Mb (%)	Predicted single-copy reads	Length in Mb	No. of contigs and singletons	Length in Mb	No. of SNPs	Nucleotides/SNP	No. of annotated genes
*Genes*									

Characterized	948,379	380.0 (8.0%)	734,848	298.8	378,152	153.4	153,787	997	32,307

Uncharacterized	285,529	113.7(2.3%)	109,158	44.0	45,570	18.1	41,844	432	

Sub total	1,233,908	493.7 (10.3%)	844,066	342.8	423,722	171.5	195,631	876	32,307

*Repetitive sequences*									

Characterized repeats	7,121,948	2,818.1 (59.0%)							

Repeat junctions^(b)^	347,811	156.9(3.3%)	200,564	89.3	200,564	89.3	145,907	612	

Sub total	7,121,948	2,818.1 (59.0%)							

*Uncharacterized sequences*	3,929,444	1,460.7 (30.7%)	2,398,762	891.2	1,236,912	271.5	155,580	1745	

Total	12,285,300	4,772. 5 (100%)	3,443,392	1,323	1,861,198	532.3	497,118	1070	32,307

(a) Number of reads after removing chloroplast and mitochondrial reads and artificial replicates using the cd-hit-454 program [27] at 98% alignment identity and 90% of sequence coverage. (b) Repeat junctions are identified from characterized repeat sequences using the repeat junction annotation pipeline program (Table S1 in Additional file 1).

After removing characterized repeats, gene annotation was performed. A total of 948,379 gene-related reads were detected, which is 8.0% of a total of 12,285,300 reads (the total reads are the number of reads after the removal of chloroplast and mitochondrial reads and artificial duplicates, Table 4). In order to identify unknown gene reads, SOLiD cDNA reads were mapped to the Roche 454 reads. A total of 285,529 unknown gene reads (2.3% of the total reads) were obtained. Combined with characterized gene reads, 1,233,908 reads were gene-related reads, accounting for 10.3% of the total reads. Thus, both gene and repeat reads accounted for 69.3% of the total reads. The remaining 30.7% were uncharacterized reads, which contained single-copy and multi-copy reads. Single copy read prediction was comparable on different sequencing platforms. Using the single-copy read prediction method, 347,199, 281,513 and 97,399 single-copy reads were identified in Roche 454 reads of AL8/78 with SOLiD, Solexa, and Roche 454 genomic reads of AS75, respectively (Table 5). Contingency χ ^2 ^tests showed that despite low genome coverage of Solexa or Roche 454 reads, the single-copy prediction method yielded comparable numbers of single-copy reads. A total of 844,066 of the 1.35X Roche 454 reads were predicted to be single-copy gene reads, 200,564 reads were RJs and 2,398,762 reads were single-copy reads from uncharacterized regions (Table 4).

**Table 5:** Comparison of single-copy read predictions by different sequencing platforms mapped to characterized gene contigs (including singletons) of *Ae. tauschii *accession AL8/78 sequenced with Roche 454

Sequencing platform and DNA source	Roche 454 contigs mapped	**Contigs shared with SOLiD**^**(a)**^	Cut-off value for single-copy prediction	Single- copy Roche 454 contigs (% of mapped contigs)	**Single-copy contigs shared**^**(a)**^	**Contingency χ **^**2 **^**test *P *value**
SOLiD (~10.7X)^(b)^	378,185		53	347,199 (91.8%)		

Solexa (~2.1X)	305,495	299,673	8	281,513 (92.2%)	260,261 (86.8%)	0.0001

Roche 454 (~1.6X)	104,030	101,800	8	97,399 (93.6%)	78,963 (77.6%)	0.0001

(a) Number of Roche 454 contigs to which reads generated with an indicated platform and SOLiD were mapped. (b) The coverage estimate is the average depth of sequences mapped to Roche 454 contigs.

### Genome-wide SNP discovery and characterization

SNPs were discovered with the SNP discovery pipeline using the predicted Roche 454 single-copy reads in genes, RJs and uncharacterized regions as a reference sequence (Figure 4 and Table S1 in Additional file 1). A total of 195,631 SNPs were discovered in gene regions, which included 153,787 and 41,844 SNPs in characterized and uncharacterized gene regions, respectively (Table 4 and 6). In addition, 145,907 SNPs were discovered in repeat junctions and 155,580 in uncharacterized regions (Table 4). Relatively more SNPs were in repeat junctions (one SNP per 612 bp) than in genes (one SNP per 876 bp). The SNP frequency in uncharacterized regions cannot be compared with those in repeat junctions and genes because more stringent criteria were applied to SNP discovery in those regions (see Materials and Methods).

**Figure 6:**
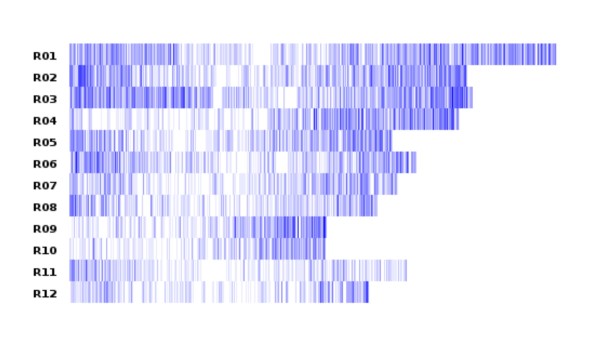
**The abundance and distribution of rice genes homologous to *Ae. tauschii *genes bearing SNPs across the 12 rice chromosomes**. Each heat map track represents a rice chromosome from R01 to R12. The range of number of rice genes homologous to *Ae. tauschii *genes with SNPs in a bin with a bin width of 0.1 Mb is from 0 (white color) and 11 (deepest blue color).

Of the 153,787 SNPs in characterized genes, 99,697 SNPs were in gene sequences that showed BLAST homology to 27,459 different wheat unigenes, 9,400 to rice genes, and 13,728 to *Brachypodium distachyon *genes, covering a total of 32,307 different genes. A total of 7,479 SNPs were in sequences homologous to 3,565 bin-mapped wheat ESTs with an average of 137 SNPs per bin and a standard deviation of 96 SNPs. Sequences with 69,125 SNPs were homologous to genes in the *Brachypodium *genome yielding 255 SNPs per Mb of the *Brachypodium *genome. Sequences harbouring 20,386 SNPs were homologous to genes in the rice genome yielding 53 SNPs per Mb of the rice genome. Gene sequences harbouring SNPs in *Ae. tauschii *were distributed across the entire rice genome (Figure 6), suggesting that those SNPs were also distributed cross the entire *Ae. tauschii *genome.

Different NGS platforms and DNA sources can be used for SNP discovery (Table 6). The SNP discovery pipeline contains two major steps implemented in two separate programs (Figure 3 and Table S1 in Additional file 1): read mapping and SNP calling (bwa_snp_pipeline.pl), and SNP filtering (snp_filter_pipeline.pl). The read mapping and SNP calling are performed separately for data of each individual sequencing platform. However, the SNP filtering procedure can use either merged SNPs called from all available platforms or SNPs called from one sequencing platform at a time. In this study, we performed SNP discovery from sequences of three NGS platforms. If the merged SNPs called from three sequencing platforms were used for SNP filtering, a total of 195,631 putative gene-related SNPs were obtained, but if SNP filtering was carried out separately for each sequencing platform, only a total of 83,531 putative gene-related SNPs (42.7%, less than a half of SNPs identified from the merged data) were detected (Table 6), suggesting that combining sequence data from multiple NGS platforms will help increase SNP discovery rate. This is because for many sequence regions, the mapped read depth was low (< 3 reads) in a single sequencing platform and no SNPs can be called according to the SNP filtering criteria. Merged alignment data from several sequencing platforms increased mapped read depth and thus more SNPs can be identified.

**Table 6:** SNP discovery in *Ae. tauschii *genes using different sequencing platforms and DNA sources

**Sequencing platform(DNA source)**^**(a)**^	SNPs	SNPs %	Number of reference sequences with SNPs (AL8/78)	Reference sequences with SNPs %
*Group by combinations of sequencing platforms and DNA sources (SNP filtered with merged SNPs discovered by three sequencing platforms)*				

Roche 454(genomic)	17,228	8.81	10,199	6.69

Solexa(genomic)	17,434	8.91	14,130	9.27

Solexa(genomic)/Roche 454(genomic)	960	0.49	863	0.57

SOLiD(cDNA)	36,667	18.75	31,963	20.98

SOLiD(cDNA)/Roche 454(genomic)	106	0.05	105	0.07

SOLiD(cDNA)/Solexa(genomic)	879	0.45	860	0.56

SOLiD(cDNA)/Solexa(genomic)/Roche 454(genomic)	21	0.01	21	0.01

SOLiD(genimc)/Solexa(genomic)/Roche 454(genomic)	95	0.05	92	0.06

SOLiD(genomic)	102,902	52.60	76,267	50.05

SOLiD(genomic)/Roche 454(genomic)	424	0.22	413	0.27

SOLiD(genomic)/Solexa(genomic)	3,567	1.82	3,280	2.15

SOLiD(genomic)/SOLiD(cDNA)	14,968	7.65	13,812	9.06

SOLiD(genomic)/SOLiD(cDNA)/Roche 454(genomic)	19	0.01	19	0.01

SOLiD(genomic)/SOLiD(cDNA)/Solexa(genomic)	351	0.18	348	0.23

Total	195,631	100.00	152,372	100.00

*Group by single sequencing platform or DNA source (SNP filtered with merged SNPs discovered by three sequencing platforms)*				
SOLiD(genomic) (~10.7 X)^(b)^	122,326	62.53	94,231	61.84

SOLiD(cDNA) (~21.5X)	53,021	27.10	47,128	30.93

Solexa(genomic) (~2.1X)	23,307	11.91	19,594	12.86

Roche 454(genomic) (~1.6X)	18,853	9.64	11,712	7.69

Total^(c)^	217,507	111.18	172,665	113.32

*SNPs filtered by individual sequencing platforms*				

SOLiD(genomic) (~10.7 X)^(b)^	55,657	72.67	50,639	76.50

SOLiD(cDNA) (~21.5X)	17,935	23.42	16,726	25.27

Solexa(genomic) (~2.1X)	7,729	10.09	7,323	11.06

Roche 454(genomic) (~1.6X)	2,210	2.89	1,979	2.99

Total^(d)^	83,531	109.07	76,667	115.83

(a) Three different sequencing platforms (Roche 454, Solexa and SOLiD) and two DNA sources (Genomic DNA and cDNA) were used in SNP discovery. SNPs were counted by combinations of sequencing platforms and DNA sources or individual sequencing platform/DNA sources. (b) Genome coverage in parentheses was estimated from the length of mapped gene reference sequences and mapped read depths (See Figure 2). (c) If SNPs were filtered by merged SNPs from three sequencing platforms, the total number of SNPs and reference sequences with at least one SNP are 195,631 and 152,372, respectively. Because the same SNPs may be discovered by both cDNA and genomic reads, or by more than one sequencing platforms, the sum of SNPs in grouping by single sequencing platform or DNA source exceeds the actual number of SNPs. The difference will be the number shared by two DNA sources or multiple sequencing platforms. The same is true for number of reference sequences and percentage values in the table. (d) If SNPs were filtered by individual sequencing platforms (run the entire SNP discovery pipeline by individual sequencing platforms), the total number of unique SNPs discovered and reference sequences with at least one SNP were 76,588 and 66,190, respectively. For the same reason as (c), the sum of SNPs discovered by single sequencing platform or DNA source exceeds the actual SNPs.

**Figure 7:**
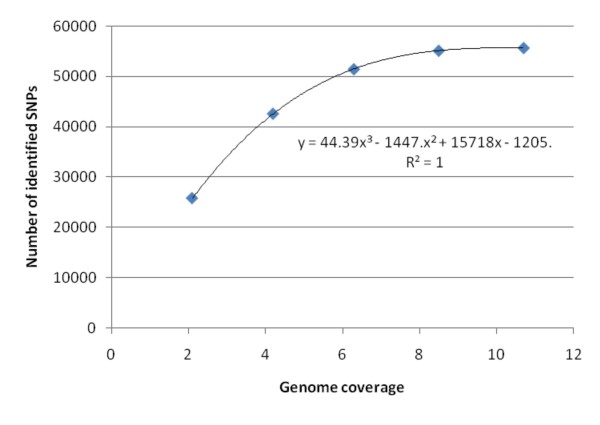
**The number of gene SNPs discovered is significantly correlated to genome coverage of reads mapped to reference sequences**. Random samples of SOLiD genomic reads with ~2X (1 run of SOLiD sequencing), ~4X (2 runs), ~6X (3 runs), ~8X (4 runs) and ~10X (5 runs) genome equivalents were used for SNP discovery. The values of genome coverage were estimated based on AS75 reads mapped to the annotated Roche 454 gene reads (Figure 2). The genome coverage of 10.7X (Table 6) based on this method is equivalent to 26.57X (Table 1) which was estimated based on the 4.02 Gb genome size of *Ae. tauschii*.

The varying numbers of SNPs were discovered with different NGS platforms because of their varying genome coverage (Table 6). Genome coverage of reads was significantly correlated with the numbers of SNPs discovered with a NGS platform (Table 6 and Figure 7).

The same SNPs identified by two or more sequencing platforms account for 3.3% (6,422 SNPs) of all putative gene-related SNPs (Table 6). The percentage of the same SNPs identified by two sequencing platforms should be associated with overlapping percentage of reads between two sequencing platforms, which depends on genome coverage of reads obtained from sequencing. Simulation results showed that if the percentage of the same SNPs in two sequencing platforms is 3.3%, the overlapping percentage of reads generated from two sequencing platforms must be over 12%. If this overlapping percentage reaches to 80%, the percentage of the same SNPs will be 79% (Figure S1 in Additional file 1). Twice as many SNPs were identified using SOLiD sequencing of genomic DNA than cDNA, which is not surprising since SNP discovery in cDNA is limited by the number of genes sampled by the cDNA library.

### Validation of *Ae. tauschii *SNPs

In order to assess the veracity of discovered SNPs and estimate false-positive SNP discovery rate, 192 gene sequences with at least one SNP were randomly chosen from SNP reference sequences with a dataset of 10,000 gene-based SNPs used for Illumina Infinium genotyping (Table 7). SNP flanking primers were designed with BatchPrimer3 [35]. Only 130 of 192 primer pairs generated PCR products in both AL8/78 and AS75. Failure to amplify target DNA by some primer pairs was primarily due to unoptimized PCR conditions which was confirmed by using the optimized PCR conditions in the second run of PCR amplification for one plate of these primers. The products were sequenced with an ABI 3730 xl and sequences were aligned. The 130 aligned regions were expected to contain 187 putative SNPs discovered with the pipeline, of which 157 SNPs were present. Hence, the SNP validation rate was 84%. Of 30 false positive SNPs, 25 (83%) were due to SOLiD or Solexa consensus sequencing errors or to incorrect alignments and only 5 SNPs (17%) were due to Roche 454 sequencing errors (Table 7).

**Table 7:** PCR validation of *Ae. tauschii *SNPs

Category	**Sequenced loci**^**(a)**^	Total SNPs	Validated SNPs	False positive SNPs	False positive SNPs due to reference	False positive SNPs due to mapped reads	SNP validation rate %
*Sequence category*							

Genes	130	187	157	30	5	25	84.0

Repeat junctions	52	67	59	8	4	4	88.0

Uncharacterized	43	48	39	9	5	4	81.3

Total	225	302	255	47	14	33	84.4

*Sequencing platforms*							

AB SOLiD	181	246	217	29	11	18	88.2

Roche 454	19	35	25	10	2	8	71.4

Illumina Solexa	84	96	82	14	6	8	85.4

Total^(b)^	284	377	324	53	19	34	85.9

(a) Only loci with PCR products and Sanger sequences in both *Ae. tauschii *accessions (AL8/78 and AS75) were included in the table. One primer pair for each locus was designed for PCR amplification. (b) Since some SNPs were identified by more than one sequencing platform, the total numbers of SNPs therefore exceed 302.

A total of 96 RJ sequences were randomly chosen from the predicted single-copy RJ sequences with a SNP within 50 bp of RJ location. Target DNA at 24 RJ sequences did not amplify in PCR. Of the remaining 72 RJ targets, 20 amplified either only the AL8/78 DNA target (15 out of 20) or had no alignments in the SNP locations (5 out of 20) reflecting diversity between AL8/78 and AS75 in repeated sequences. The remaining 52 RJ targets could be amplified in both DNAs and were expected to contain 67 putative SNPs in the aligned regions. Of these 67 putative SNPs, 59 (88%) were present (Table 7). The SNP validation success rate in RJs was similar to that in gene sequences, showing that single-copy RJs are a productive source of useful SNPs.

Similarly SNPs discovered in uncharacterized sequences were also verified. A subset of 95 uncharacterized sequences were randomly sampled from the reference sequences with 155,580 uncharacterized SNPs. Out of 95 sequences, 18 sequences did not amplify in both accessions (AL8/78 and AS75) and 34 sequences amplified only in one of two accessions or their target sequences had no alignments in the SNP locations. The high failure rate of PCR amplification is likely due to diversity between AL8/78 and AS75 in uncharacterized regions because most of SNPs in uncharacterized sequences should be located in non-coding regions. The remaining 43 sequences amplified in both DNAs were expected to have 48 putative SNPs in the aligned regions. Of these 48 putative SNPs, 39 (81%) were validated (Table 7). The SNP validation rate in the uncharacterized regions was slightly lower than that in gene and RJ sequences.

SNP validation rates associated with individual NGS platforms were assessed (Table 7). SOLiD and Solexa had similar SNP validation rates (88.2% and 85.4%, respectively). Validation of putative SNPs discovered by mapping Roche 454 reads to Roche 454 reference sequence revealed a 71% SNP validation rate. The most likely cause of the lower rate associated with Roche 454 was the shallow depth of Roche 454 read mapping.

A set of SNPs between *Ae. tauschii *accessions AL8/78 and AS75 was previously discovered by Sanger sequencing of single-copy genes [37]. A total of 1,212 SNPs located in 641 genes were genotyped with the Illumina GoldenGate SNP assays and mapped on an *Ae. tauchii *genetic map [18]. Of the 641 genes, 192 shared sequence with NGS genic sequences generated here. There were 223 SNPs in these 192 genes, of which 161 (72.2%) were shared by both data sets, indicating they can be genotyped with Illumina GoldenGate assays and mapped.

## Discussion

### Annotation-based genome-wide SNP discovery pipeline, AGSNP

We report here the development of a pipeline for large-scale, genome-wide SNP discovery in large and complex genomes with NGS platforms. This pipeline does not require a reference genome sequence, and its utility is illustrated using the 4.02 Gb genome of *Ae. tauschii*. The large volume of NGS data that must be processed places great demands on computer resources. The pipeline was therefore split into multiple sub-pipelines to perform individual tasks and accomplish its two principal objectives. The first objective is the assignment (annotation) of Roche 454 reads of a single genotype to three categories: (1) characterized gene reads, (2) characterized repeats, and (3) uncharacterized reads. The second objective is predicting single-copy reads and identifying the putative SNPs by mapping multiple genome equivalents of SOLiD, Solexa or Roche 454 reads to the annotated, single-copy Roche 454 reads. The use of single-copy reads in the reference sequence dramatically reduces data processing and computation time to a manageable amount during the SNP discovery phase.

An asset of the pipeline is that it employs computational tools for read mapping and SNP calling that are applicable to any NGS platform. The pipeline is consequently of a universal utility with the existing and future NGS platforms. The Roche 454 platform used here to generate long reads for the construction of the reference sequence can be replaced by any platform that produces reads of a comparable or greater length, particularly if it would have higher throughput than the Roche 454. Flexible and stringent SNP filtering criteria implemented in the pipeline result in the discovery of large numbers of SNPs and low false-positive SNP rates. A total of 497,118 SNPs was identified, of which 195,631 were in genes. SNPs in genes had an 84% validation rate, those in RJs had an 88% validation rate, and those in uncharacterized sequences had 81% validation.

In the pipeline, a reference sequence of relatively shallow genome coverage of one genotype is compared with reads of another genotype with deep coverage. The latter reads can be short, and although any of the current NGS platforms can in theory be used, the overriding requirement is that the platform has a very high throughput to minimize sequencing costs. This requirement is particularly critical for large and complex genomes, such as that of *Ae. tauschii *or related wheat.

The pipeline can be used to discover SNPs in both genomic DNA and cDNA. Because genomic DNA is more complex than most cDNA resources, more than twice as many SNPs were identified in genomic DNA than cDNA here. Genomic DNA is therefore preferable for SNP discovery over cDNA. Even for a genome as large as that of *Ae. tauschii*, only 2.5 runs with the SOLiD v3 were needed to generate a sufficient number of genomic reads to control error during SNP discovery. A single run of SOLiD v4 would be needed to achieve the same coverage. An additional disadvantage of using data of cDNA alone, in addition to the labour associated with the construction of a cDNA library, is that it does not facilitate the annotation of uncharacterized single-copy sequences in the reference sequence; only genes can be used for SNP discovery. Therefore, the use of cDNA for SNP discovery limits the total amount of DNA used for SNP discovery and hence the total number of SNPs discovered.

Another advantage of using genomic DNA for SNP discovery is access to RJs, which are an important source of polymorphisms. SNPs were 1.7 times more frequent per kb in RJ than in genic regions, while having equally high validation rate. Higher polymorphism in RJ makes them particularly valuable for plants with generally low levels of SNP. The RJPrimers program used in one of the sub-pipelines in the AGSNP pipeline, facilitates the identification of single-copy repeat junctions. SNPs in repeat junction regions can by genotyped in a high-throughput mode, e.g., with Illumina's GoldenGate assay [11], which makes them a valuable marker system.

### Error sources during SNP discovery with NGS platforms

Errors in SNP discovery have two major sources: (1) sequencing errors and (2) errors in mapping of short reads to Roche 454 reference sequence. The sequencing errors for NGS platforms are less than 1%. The vast number of sequencing errors in all three NGS platforms is INDELs [38]. Filtering INDELs and homopolymers and the use of multiple genome equivalents can reduce sequencing error rate [39]. The base substitution error rates in consensus sequences of Solexa and SOLiD were very low, about four bases in 10,000. The combined error rate of the Roche 454 reference sequence and mapped reads were 0.03%, 0.15% and 0.27% for SOLiD, Solexa, and Roche 454. Therefore, since sequencing errors are an insignificant source of false-positive SNP rates, the major source of SNP errors is mapping errors. The use of single-copy reads in the pipeline helps to reduce those errors.

The validation rate of RJ SNPs (88%) was as high as that of gene SNPs (84%). All RJ SNPs used for validation were randomly selected from a set of the predicted single-copy RJ sequences with a SNP within 50 bp of RJ locations, which are Illumina genotyping-ready. Previous study indicated that in the Illumina GoldenGate genotyping assays the success rate was higher when a repeat junction was in the vicinity of the target SNP as compared with RJ SNPs without a repeat junction [11]. RJ SNPs in vicinity of repeat junctions must also have a high SNP validation rate and should therefore be selected as a first priority for RJ SNP markers.

The 84%, 88% and 81% SNP validation rate for genes, RJs and uncharacterized regions achieved here with genomic DNA is comparable to that reported by others with NGS of cDNA libraries; an 83% SNP validation rate was reported for *Eucalyptus grandis *cDNA sequenced with Roche 454 [40], an 85% validation rate was achieved in maize cDNA sequenced with Roche 454 [7], and an 87.4% validation rate was reported in *Brassica napus *cDNA sequenced with Solexa [7]. Our validation rate was somewhat lower than those reported for SNPs discovered in RRL. Validation rates of 79%-92.5% were reported in soybean RRL sequenced with Solexa [5], 96.4% and 97.0% validation rates were respectively reported in rice and soybean RRL sequenced with Solexa [4], and an 86% validation rate was reported for RRL library in common bean sequenced with a combination of Roche 454 and Solexa [6]. However, complete reference genomes were used in those SNP discovery projects. In addition, none of these genomes is equal in size and complexity to the genome of *Ae. tauschii*, which underscores the general utility of the AGSNP pipeline, its high SNP discovery rate, and its particular utility for SNP discovery in large and complex genomes, such as those of many plants.

### SNP discovery in *Ae. tauschii*

A total of 195,631 SNPs in genes and 145,907 SNPs in repeat junctions were discovered in this study. The SNP frequencies were one SNP per 876 bp for genes, and one SNP per 612 bp for repeat junctions, respectively. Repeat junctions have a higher SNP frequency than genes, which is consistent with the results from Paux et al. (2010) [11]. But this is still lower than the frequency observed previously in coding regions of wheat with ranges from one SNP per 267 bp [41] to one SNP per 540 bp [42], or in the coding region of *Ae. tauschii *with one SNP per 202 bp [37]. In the genic regions of *Ae. tauschii*, nucleotide polymorphism was estimated to be 2.44 × 10^-3 ^[43], which is equivalent to one SNP every 409 bp between two randomly selected haplotypes. The expected SNP frequency in genes is therefore at least 2.1-fold higher than that obtained in the SNP discovery here (one SNP every 876 bp in genes). Taking into account the fact that the accession AL8/78 and AS75 were not selected in random - they were selected because they differed greatly on the basis of RFLP [18] - the number of SNPs discovered in this project, although very high, is fully realistic for these two accessions.

Several major factors impact genome-wide SNP discovery in the *Ae. tauschii genome *using next generation sequencing and account for the fact that only about one half of genic SNPs expected were discovered. (1) Low genome coverage (~1.35X) of Roche 454 sequences of one genotype AL8/78 was used as reference sequences. According to simulation results from 13 *Ae. tauschii *BACs (Figure S2 in Additional file 1), ~70% of gene sequences are covered at ~1.5X genome coverage of Roche 454 reads. At least 3X coverage genome equivalents are required for over 90% coverage of gene sequences (Figure S2 in Additional file 1). (2) The second factor is genome coverage of mapping reads sequenced in another genotype (AS75). The total number of discovered SNPs is significantly correlated with genome coverage of mapping reads (Figure 7). Increasing genomic coverage of mapping reads can increase coverage percentage and mapped read depth to a reference sequence, resulting in the increase in SNP discovery rate. (3) The last factor, which is of general significance, is the number of diverse lines used for SNP discovery. In this study, only two genotypes were used. Simulation results with simplified assumptions showed that over 90% of expected number of SNPs can be discovered when more than 5 genotypes are sequenced (Figure S3 in Additional file 1). This fact should be taken into account in projects targeting species-wide SNP discovery.

## Conclusions

We demonstrated here that high numbers of genome-wide SNPs can be discovered by sequencing total genomic DNA of a complex genome with NGS platforms without a reference genome sequence. Using the AGSNP pipeline, 195,631 putative SNPs in genes, 145,907 putative SNPs in repeat junctions and 155,580 putative SNPs in uncharacterized reads were discovered in genomic sequences of two accessions of *Ae. tauschii*. The SNP validation rates obtained here were comparable to those obtained with the cDNAs of less complex plant genomes. The strategy described here and the associated pipeline yielded more SNPs while being otherwise comparable to cDNA or RRL approaches.

## Availability and requirements

Project name: Annotation-based genome-wide SNP discovery pipeline

Project home page: http://avena.pw.usda.gov/wheatD/agsnp.shtml

Availability: Freely available

Operating systems: Linux

Programming language: Perl and Java

Other requirements: bwa, SAMTools, gsAssembler (Newbler), cd-hit-454

License: GNU PGL

Any restrictions to use by non-academics: None

## Authors' contributions

FMY, YQG, MCL, DL, PEM, JD, and ODA planed the work. NH and YQG performed Roche 454 sequencing and KRD, MCL, and JD performed SOLiD sequencing. Solexa sequencing was performed in UC Davis Genome Center. NH, YQG, and KRD performed BAC sequencing with the Sanger method. FMY performed pipeline development, data analysis and SNP discovery. NH and YQG performed SNP validation. FMY and JD drafted the manuscript. All authors read and approved the final draft of the manuscript.

## Supplementary Material

Additional file 1 **Supplementary tables and figures**. The file contains Table S1, Figure S1, S2 and S3. Table S1 lists all pipeline scripts for annotation-based SNP discovery. Figure S1 shows the relationship of percentage of the same SNPs identified by two NGS platforms with overlapping percentage of reads generated between two NGS platforms. Figure S2 presents gene coverage of NGS reads with different genome coverage of NGS reads and with different sequencing platforms. Figure S3 depicts the relationship of the number of detected SNPs with the number of genotypes used for SNP discovery. Click here for file
