# Supplementary material for: Annotation-based genome-wide SNP discovery in the large and complex Aegilops tauschii genome using next-generation sequencing without a reference genome sequence
Source: BMC Genomics. 2011 Jan 25;12:59. doi: 10.1186/1471-2164-12-59 (PMC3041743; doi:10.1186/1471-2164-12-59)
Supplement: Additional file 1 — Supplementary tables and figures. The file contains Table S1, Figure S1, S2 and S3. Table S1 lists all pipeline scripts for annotation-based SNP discovery. Figure S1 shows the relationship of percentage of the same SNPs identified by two NGS platforms with overlapping percentage of reads generated between two NGS platforms. Figure S2 presents gene coverage of NGS reads with different genome coverage of NGS reads and with different sequencing platforms. Figure S3 depicts the relationship of the number of detected SNPs with the number of genotypes used for SNP discovery. [file 1471-2164-12-59-S1.PDF]

Table S1: Pipeline programs of annotation-based SNP discovery

Function	Program	Dependency
Roche 454 read annotation		
Batch BLAST	multi-blast.pl<br>batch_blast2table.pl<br>unique_query_ids.pl<br>extract_seqs.pl	Blast2 package
Gene annotation	bwa_mapping_pipeline.pl	bwa package
Repeat annotation	repeat_junction_annotation.pl	RJPrimers
Assembly	batch_gsassembly.pl	gsAssembler
Removing artificial duplicates	batch_clustering_reads.pl	cd-hit-454
SNP discovery		
Format conversion utilities	roche2fastq.pl<br>fasta2fastq.pl<br>solid2fastq.pl	bwa package
Read mapping and SNP calling	bwa_snp_pipeline.pl	bwa package<br>SAMTools
SNP filtering	summarize_bwa_snp_calls.pl<br>snp_filter_pipline.pl	
SNP selection for Illumina assay design	snp_selection_for_asssay_design.pl	

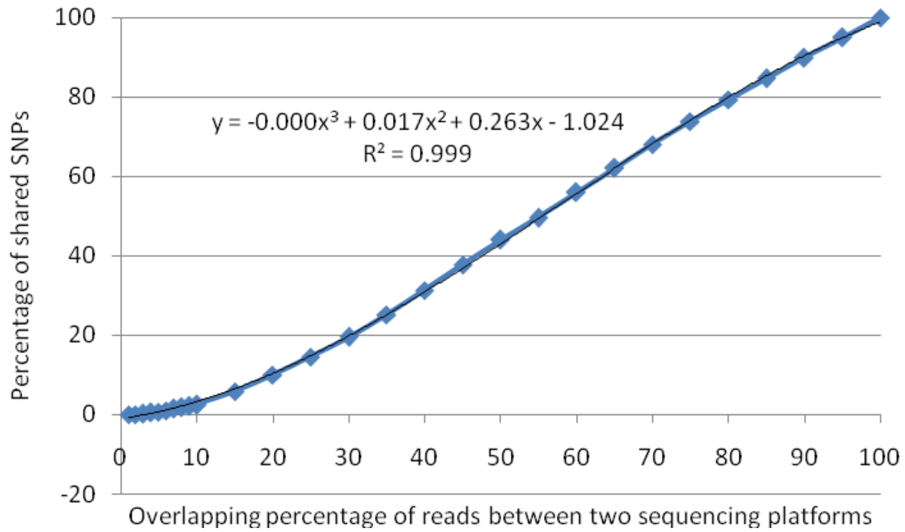

Figure S1. Relationship of percentage of the same SNPs identified by two NGS platforms with overlapping percentage of reads generated between two NGS platforms. The percentage of the same SNPs by two sequencing platforms should be associated with overlapping percentage of reads generated from two sequencing platforms which depends on genome coverage of sequenced reads of each sequencing platform. Data was simulated based on the following simplified assumptions: (1) the same amount of sequencing data are generated by three sequencing platforms (Roche 454, Solexa and ABI SOLiD (2) two genotypes are used for SNP discovery; and (3) reference sequences (Roche 454 reads here) cover all SNPs.

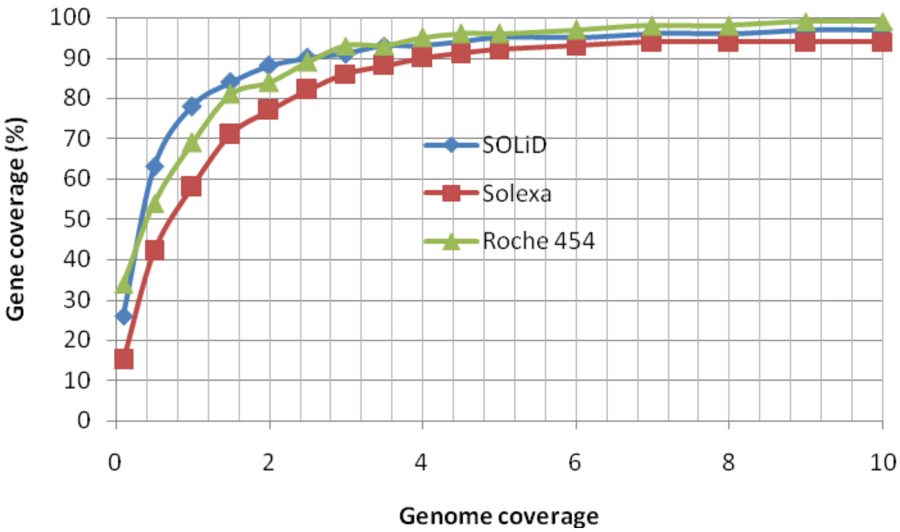

Figure S2. Gene coverage of NGS reads with different genome coverage of NGS reads and with different sequencing platforms. Thirteen BACs of AL8/78 were sequenced with three NGS platforms and Sanger technology (Table 1). Sanger sequences of 13 BAC were annotated and 38 genes with 73,015 bp in length were identified. NGS Reads were randomly sampled for each sequencing platform and for different genome coverage. Sampled NGS reads with different genome coverage were mapped to 38 gene sequences. Gene coverage was calculated by mapped gene length divided by total gene length. Simulation results showed that gene coverage of NGS reads slightly varied with different sequencing platforms but significantly changed with increasing genome coverage. For example, for the Roche 454 sequencing platform, ~70% of gene sequences can be covered at 1.5X genome coverage, whereas over 90% of gene sequences can be identified at 3X genome coverage of reads.

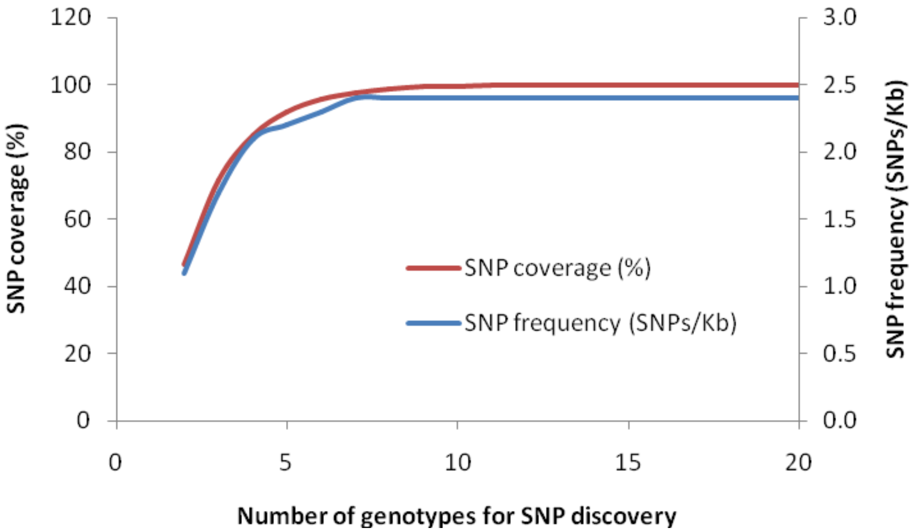

Figure S3. Relationship of the number of detected SNPs with the number of genotypes used for SNP discovery. Data was simulated based on the following simplified assumptions: (1) The nucleotide polymorphism rate of *Ae. tauschii* for coding region was estimated to be 2.44×10^{-3} (Dvorak et al. 2010 [1]), which is equivalent to one SNP every 409 bp between two randomly selected haplotypes. Therefore, the total expected SNPs of gene regions in *Ae. tauschii* can be estimated as 983,320 (4.03GB × 10% of gene sequences × 2.44×10^{-3} of substitution rate). (2) All genotypes have similar nucleotide diversity. SNP frequency for gene regions from two genotypes used in this study was estimated to be one SNP per 876 bp (Table 4). This estimate is applied to all genotypes although different genotypes vary in nucleotide diversity. (3) All genotypes are independently sequenced using next generation sequencing technologies in the similar genome coverage. SNP coverage (%) is calculated by number of identified SNPs divided by the total expected SNPs.

References

1. Dvorak J, Luo MC, Akhunov ED: **N.I. Vavilov's theory of centers of diversity in the light of current understanding of wheat domestication and evolution**. In: *The 8th International Wheat Conference 2010; St. Ptersburg*: Czech J Genetics and Breeding; 2010 (in press).
